# IDH1 mutation creates a dependency on fatty acid metabolism that underlies sensitivity to cuproptosis in acute myeloid leukemia cells

**DOI:** 10.7150/ijms.127886

**Published:** 2026-02-26

**Authors:** Xuening Zhang, Dayuan Zheng, Tong Chu, Dongfan Yang, Kuanyun Zhang, Shaokui Liang, Lu Yang, Wenzhe Ma

**Affiliations:** 1 State Key Laboratory of Mechanism and Quality of Chinese Medicine & School of Pharmacy, Faculty of Medicine, Macau University of Science and Technology, Macau SAR 999078, China.; 2 Zhuhai MUST Science and Technology Research Institute, Macau University of Science and Technology, Hengqin Guangdong-Macao In-Depth Cooperation Zone, Guangdong, 519099, China.; 3 Department of Hematology, The Third Affiliated Hospital, Sun Yat-sen University; Institute of Hematology, Sun Yat-sen University, Guangzhou, China.

**Keywords:** isocitrate dehydrogenase 1 mutation, acute myeloid leukemia, cuproptosis, fatty acid

## Abstract

Acute myeloid leukemia (AML) harboring IDH1 mutations presents unique metabolic vulnerabilities that remain incompletely addressed by current targeted therapies. In this study, we demonstrate that IDH1-mutant AML cells are markedly more sensitive to cuproptosis induced by the copper ionophore elesclomol (ES), compared to their wild-type counterparts. While ES impairs mitochondrial function in both cell types, transcriptomic profiling reveals that ES treatment induces a global downregulation of lipid metabolism pathways. Functional assays further show that IDH1-mutant cells rely more heavily on exogenous fatty acids and exhibit impaired *de novo* lipogenesis. Under lipid-deprived conditions, ES-induced cytotoxicity is significantly enhanced, suggesting a synthetic-lethal interaction between cuproptosis and fatty acid metabolic deficiency. *In vivo* experiments confirm that ES more effectively suppresses tumor growth in IDH1-mutant xenografts. These findings uncover a copper-dependent metabolic vulnerability and provide a rationale for exploiting cuproptosis as a therapeutic strategy in IDH1-mutant AML.

## Introduction

Mutations in isocitrate dehydrogenase 1 (IDH1) have garnered sustained attention in acute myeloid leukemia (AML). These mutations occur in 5-10% of adult AML cases, with higher frequencies in elderly patients; over 90% of cases involve the R132 residue 1. Biologically, IDH1 mutations are early clonal events that promote a stem-like and inflammatory phenotype. They frequently co-occur with DNMT3A, SRSF2 and NPM1 mutations, and their dynamic allele burden reflects disease progression 2, 3. Clinically, the IDH1R132H mutation is associated shorter event-free survival 4. Mechanistically, the neomorphic activity of IDH1R132H converts α-ketoglutarate (α-KG) into the oncometabolite 2-hydroxyglutarate (2-HG), which disrupts epigenetic regulation and impairs differentiation, thereby driving leukemogenesis 5.

Currently, targeted therapy for IDH1-mutated AML focuses on two FDA-approved small-molecule inhibitors. Ivosidenib (AG-120) blocks 2-HG production by mutant IDH1 (R132C/R132H), promoting leukemic cell differentiation. It is approved as monotherapy or in combination with azacitidine for newly diagnosed patients ineligible for intensive chemotherapy 6, 7. Olutasidenib, with a similar mechanism, has recently been approved for relapsed/refractory (R/R) IDH1-mutated AML 8. However, only 30%-40% of patients achieve meaningful responses, and complete remission rates remain low 9. Resistance mechanisms include secondary IDH1 mutations (e.g., R132C/S280F), activation of bypass pathways, and clonal heterogeneity stemming from co-occurring mutations such as NPM1 10, 11. Consequently, overcoming resistance in IDH1-mutated AML remains a critical challenge.

Cuproptosis is a newly discovered form of copper-dependent programmed cell death. Overload intracellular copper binds directly to lipoylated dihydrolipoamide S-acetyltransferase (DLAT), inducing protein oligomerization. This process also leads to the loss of iron-sulfur (Fe-S) cluster proteins, triggering proteotoxic stress and cellular homeostatic disruption, ultimately resulting in cell death. Unlike apoptosis, necrosis, or ferroptosis, cuproptosis is uniquely dependent on copper ion concentration and targets lipoylated proteins directly 12.

Recent studies have highlighted the therapeutic potential of cuproptosis in oncology. Its induction significantly inhibits tumor growth in models of triple-negative breast cancer, lung cancer, and colorectal cancer 13-15. Cuproptosis also reverses acquired resistance to chemotherapy, radiotherapy, and immunotherapy 16-18. Mechanistically, it remodels the tumor immune microenvironment, activating antitumor responses such as CD8⁺ T-cell-mediated cytotoxicity, thereby reducing relapse and metastasis risk 19. Notably, cancer stem cells (CSCs) are sensitive to cuproptosis under hypoxic conditions, suggesting that this strategy may eliminate CSC subpopulations refractory to conventional therapies 20. Furthermore, combination strategies integrating cuproptosis with other cell death pathways, such as ferroptosis or pyroptosis, yield synergistic antitumor effects 15, 21. Collectively, these findings position cuproptosis as a promising avenue to overcome resistance and reactivate antitumor immunity.

Taken together, despite the establishment of the IDH1 mutation as a key therapeutic target, the inhibitors developed to date have not fully met the clinical need, underscoring the urgency for alternative strategies. Recent studies have reported therapeutic advantages of mitochondrial complex I inhibitors in IDH1-mutated AML 22, 23. In our previous research, we found that berberine, a known mitochondrial complex I inhibitor, enhanced drug sensitivity in IDH1-mutated AML cells 24. This led us to reason that other approaches targeting mitochondrial integrity, such as the recently characterized process of cuproptosis which targets the mitochondrial protein DLAT, might also represent a novel therapeutic vulnerability. Therefore, in this study, we hypothesize that cuproptosis-inducing agents, such as Elesclomol, could create a therapeutic window for targeting IDH1-mutant AML. We set out to test this by systematically evaluating the sensitivity of IDH1-mutant cells to cuproptosis and exploring the underlying metabolic basis for this potential vulnerability.

## Results

### IDH1m cells exhibit higher sensitivity to cuproptosis induction

**Figure 1:**
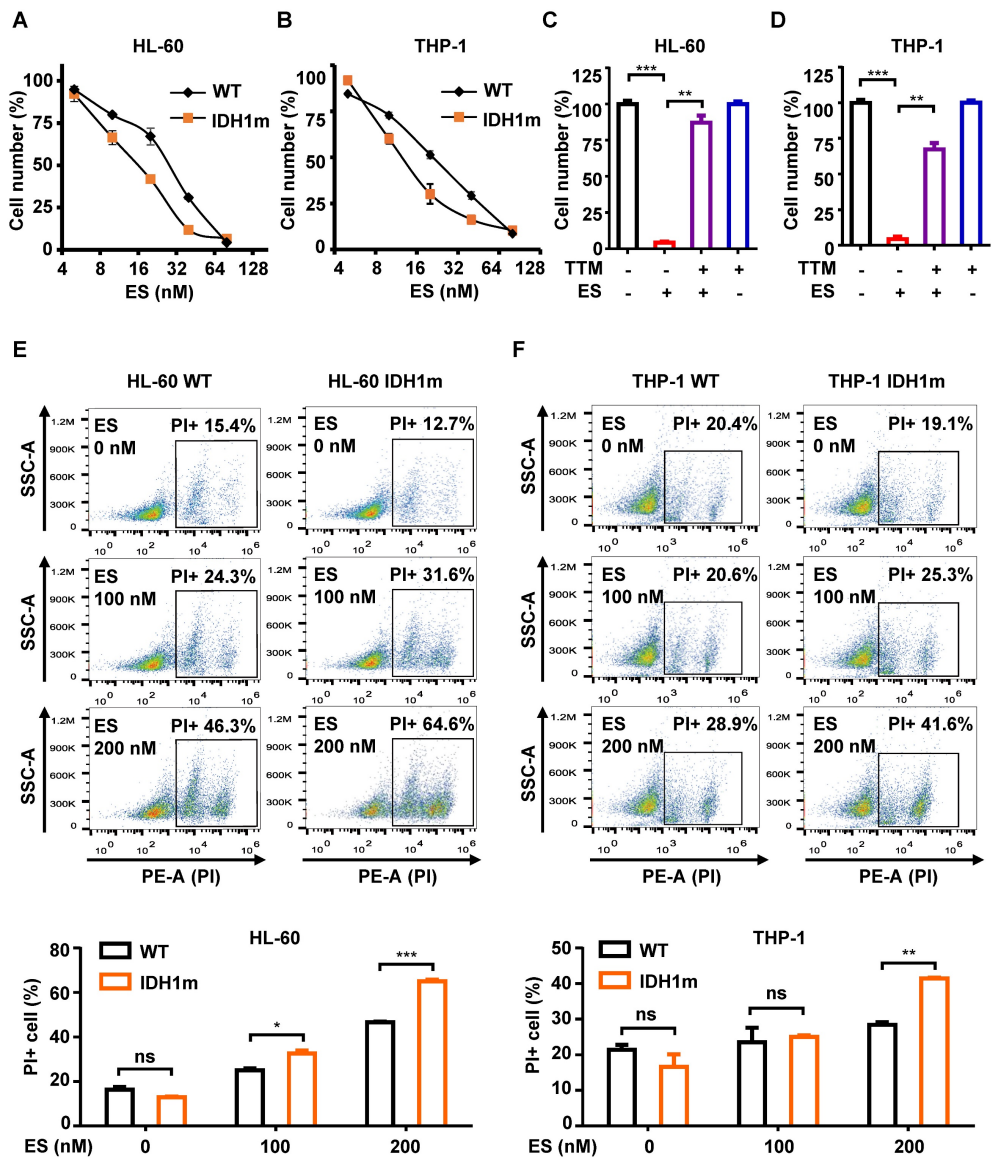
IDH1m cells exhibit higher sensitivity to the induction of cuproptosis. (A-B) ES was added to HL-60/THP-1 WT or IDH1m cells at concentrations of 0, 5, 10, 20, 40, and 80 nM for 48 h of culture; cell number was assessed by cck-8 assay. (C-D) TTM (20 µM) was added with or without ES (80 nM) to HL-60/THP-1 WT cells at the same time, and cck-8 assay was performed after 48 h of culture. (E-F) After 48 h of incubation with ES (0, 100, 200nM), the percentage of PI+ cells in HL-60/THP-1 WT or IDH1m cells was evaluated by flow cytometry. (*, *P* < 0.05; **, *P* < 0.01; ***, *P* < 0.001; ns, *P*>0.05.)

Elesclomol (ES), a potent copper ionophore and well-established cuproptosis inducer 12, was used in combination with copper chloride (1:1 ration) to induce cuproptosis. We evaluated the effects of ES on both wild-type IDH1 (WT) and R132H-mutant (IDH1m) HL-60 and THP-1 cell lines. CCK-8 assays revealed dose-dependent cytotoxicity of ES across all cell lines, with IDH1m cells exhibiting significantly enhanced sensitivity compared to WT counterparts in both HL-60 and THP-1 backgrounds (Figure 1A-1B). This cytotoxic effect was fully reversed by tetrathiomolybdate (TTM), a copper chelator (Figure 1C-1D; Figure S1A).

To further assess cytotoxicity, propidium iodide (PI) staining was used to label cells with compromised membranes. PI can only penetrate damaged membranes, making it a reliable marker of cell death. Consistent with the CCK-8 results, higher ES concentrations led to increased PI-positive cells. At equivalent ES concentrations, IDH1m cells showed a higher proportion of PI-positive cells than WT cells in both HL-60 and THP-1 lines (Figure 1E-1F).

### ES suppresses mitochondrial function comparably in WT and IDH1m cells

**Figure 2:**
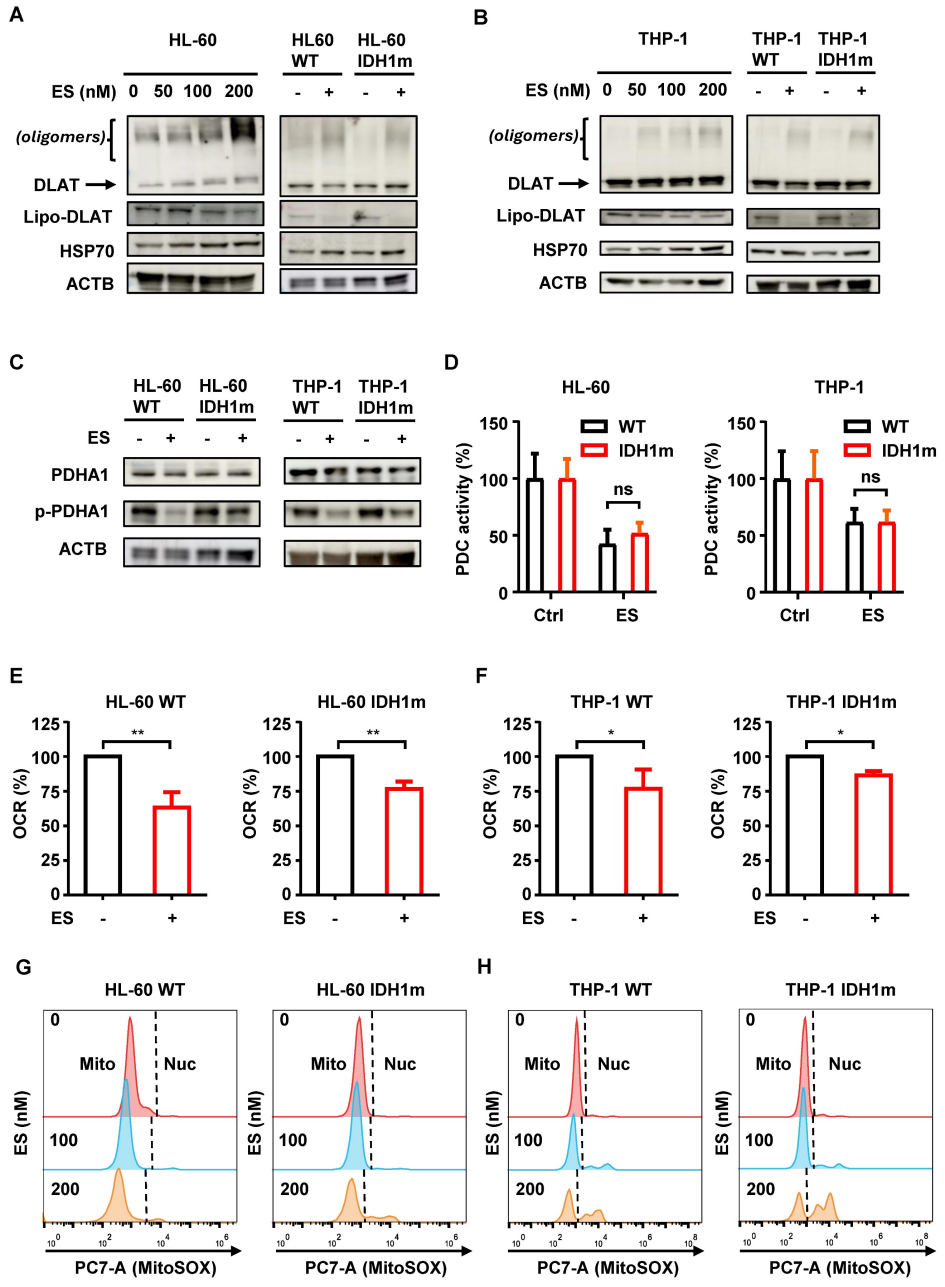
ES suppresses mitochondrial function comparably in WT and IDH1m cells. (A-B) Western blot of HL-60/THP-1 WT or IDH1m cells after 48 h treatment of 0-200 nM ES (“-” means 0 nM; “+” means 200 nM) with the indicated antibodies. (C) Western blot of HL-60/THP-1 WT or IDH1m cells after 48 h treatment of ± 200 nM ES with the indicated antibodies. (D) PDC activity measurement of HL-60/THP-1 WT or IDH1m cells after 48 h treatment of ± 200 nM ES. (E-F) The OCR was measured by the Instech Fiber Optic Oxygen Monitor System in HL-60/THP-1 WT or IDH1m cells after 24 h treatment of ± 200 nM ES. (G-H) Mitochondrial ROS measurement (“Mito” means mitochondrial signal; “Nuc” means nuclear signal) of HL-60/THP-1 WT or IDH1m cells after being treated with ES (0, 100, 200 nM) for 48 h by flow cytometry. (*, *P* < 0.05; **, *P* < 0.01.)

Cuproptosis is initiated when excess copper ions bind directly to lipoylated dihydrolipoamide S-acetyltransferase (lipo-DLAT), leading to DLAT oligomerization. This aggregation disrupts protein function and induces proteotoxic stress, which in turn activates the expression of heat shock protein 70 (HSP70) 12. Consistent with previous findings, we observed that escalating ES concentrations in HL-60 and THP-1 WT cells led to progressive DLAT oligomerization, decreased lipo-DLAT levels, and increased HSP70 expression (Figure 2A-2B). When ES was applied under identical conditions to both WT and IDH1m cells, these protein changes exhibited similar trends, with no significant differences between the two groups (Figure 2A-2B).

DLAT functions as the E2 subunit of the pyruvate dehydrogenase complex (PDC). To further investigate, we examined the E1 subunit PDHA1 and its phosphorylated (inactive) form, p-PDHA1. While total PDHA1 levels remained unchanged following ES treatment, p-PDHA1 levels were significantly reduced (Figure 2C), indicating increased PDHA1 activity. Given that DLAT oligomerization inactivates the E2 subunit and suppresses overall PDC function, the reduction in p-PDHA1 likely reflects a compensatory response to maintain PDC activity.

To validate this hypothesis, we measured PDC enzymatic activity and found that ES treatment significantly suppressed PDC function in both HL-60 and THP-1 cells. However, no significant differences were observed between WT and IDH1m cells (Figure 2D). Since PDC catalyzes the rate-limiting steP for substrate entry into the tricarboxylic acid (TCA) cycle, its inhibition provides a mechanistic basis for the observed suppression of mitochondrial respiration. As expected, ES treatment led to a significant reduction in basal oxygen consumption rate (OCR) in both HL-60 and THP-1 cells, with comparable effects between WT and IDH1m groups (Figure 2E-2F).

To assess mitochondrial reactive oxygen species (ROS), we used MitoSOX Red, a mitochondrial superoxide indicator. Increasing ES concentrations caused a leftward shift in the MitoSOX fluorescence peak, indicating a dose-dependent reduction in mitochondrial ROS levels (Figure 2G-2H). Additionally, MitoTracker DeeP Red FM (MitoT), which labels mitochondria in a membrane potential (Δψ)-dependent manner, showed a similar leftward shift with higher ES doses (Figure S1B-S1C). Since DLAT and PDHA1 protein levels remained largely unchanged (Figure 2A-2C), the reduced MitoT signal likely reflects Δψ impairment, as the dye's localization depends on intact membrane potential.

In summary, ES treatment impairs mitochondrial function in both WT and IDH1m cells, as evidenced by reduced ROS levels and diminished MitoT fluorescence. However, the extent of mitochondrial damage was comparable between the two cell types, suggesting that mitochondrial dysfunction alone does not account for the heightened sensitivity of IDH1m cells to ES.

### Transcriptomic profiling reveals that ES treatment downregulates global lipid metabolism

To investigate the metabolic consequences of ES-induced mitochondrial dysfunction, we first measured glucose uptake and lactate secretion in HL-60 and THP-1 WT cells. ES treatment significantly increased both parameters (Figure S1D-S1G), indicating compensatory activation of glycolysis 25. This metabolic shift suggests that ES may induce broader cellular reprogramming beyond mitochondrial impairment, potentially contributing to the heightened sensitivity of IDH1-mutant cells.

To explore this hypothesis, HL-60 cells were treated with 200 nM ES for 48 hours. Treated cells were designated as the “ES” group, while untreated cells served as controls (“Ctrl”). Both groups (n = 3) underwent transcriptomic profiling.

**Figure 3:**
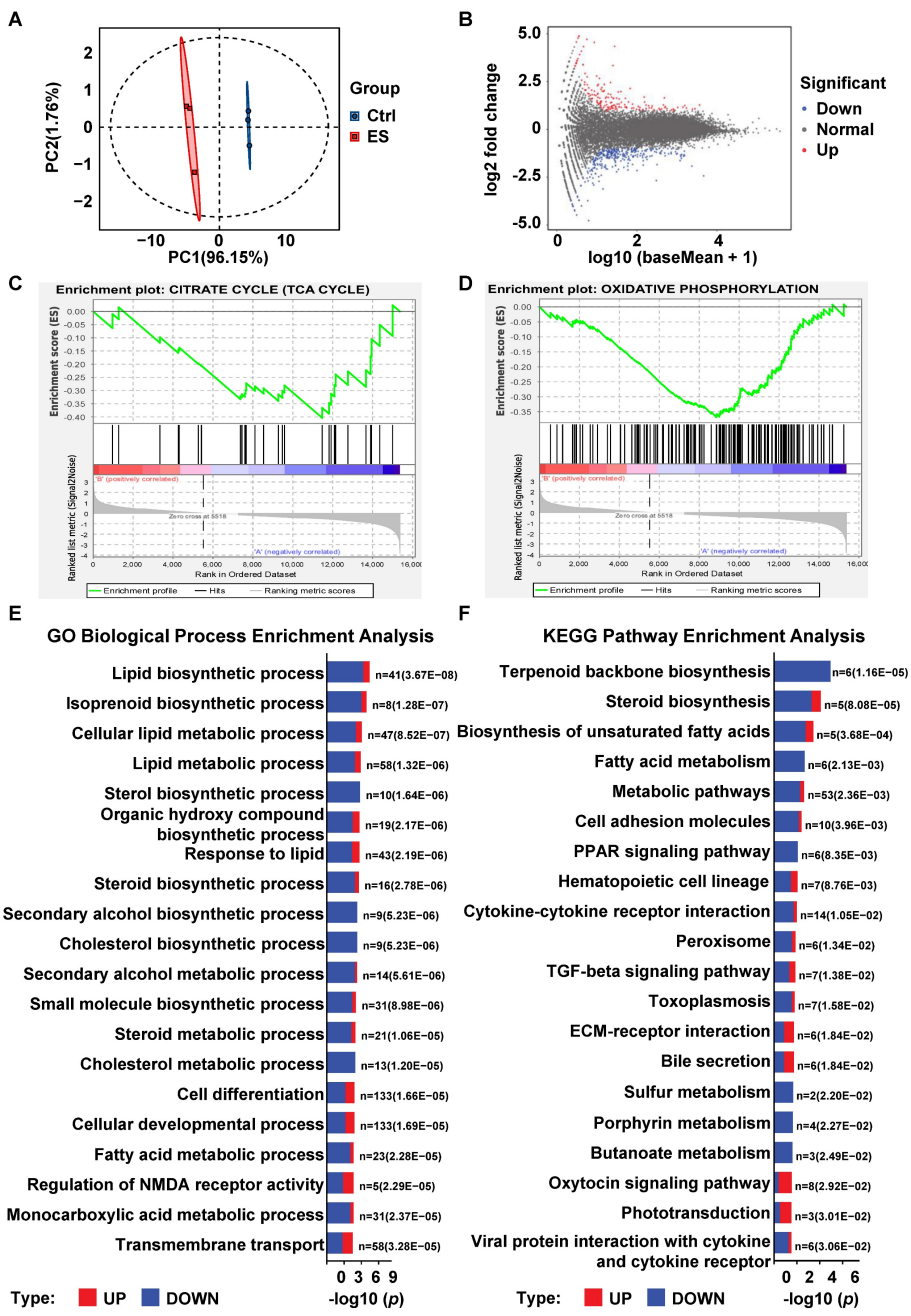
Transcriptomic profiling reveals that ES treatment downregulates global lipid metabolism. (A) The principal component analysis (PCA) of the transcriptomics data between the untreated grouP and the ES-treated grouP in HL-60 cells. (B) DEGs between the ES-treated grouP and the untreated grouP of the HL-60 cells were visualized using a MA plot (Blue dots represent genes downregulated in the ES-treated group; Red dots indicate genes upregulated in the ES-treated group; Gray dots signify genes with no significant expression differences.). (C) GSEA of DEGs comparing the ES-treated grouP to the untreated grouP of the HL-60 cells, focusing on the enrichment of the “CITRATE CYCLE (TCA CYCLE)” pathway. (D) GSEA of DEGs comparing the ES-treated grouP to the untreated grouP of the HL-60 cells, focusing on the enrichment of the “OXIDATIVE PHOSPHORYLATION” pathway. (E) ToP 20 enriched GO terms (Biological Process) for DEGs. The enrichment significance is displayed as -log10(p-value). Bars are colored to indicate the proportion of upregulated (red) and downregulated (blue) genes within each term. The corresponding p-value and gene count are annotated for each bar. (F) ToP 20 enriched KEGG pathways for DEGs. The enrichment significance is displayed as -log10(p-value). Bars are colored to indicate the proportion of upregulated (red) and downregulated (blue) genes within each term. The corresponding p-value and gene count are annotated for each bar.

Principal-component analysis (PCA) revealed clear segregation between the ES-treated and control groups, indicating substantial transcriptional changes (Figure 3A). Differentially expressed genes (DEGs) were visualized using an MA plot, with log2 fold change plotted against mean expression (log10(baseMean+1)); significantly upregulated and downregulated genes were highlighted (Figure 3B). Gene-set-enrichment analysis (GSEA) of DEGs revealed negative enrichment for “CITRATE CYCLE (TCA CYCLE)” (Figure 3C) as well as “OXIDATIVE PHOSPHORYLATION” (Figure 3D), consistent with the observed reduction in basal respiration (Figure 2E-2F).

To gain a broader view of affected pathways, we performed GO Biological Process (BP) and KEGG enrichment analyses. The toP 20 significantly enriched terms were visualized in bar plots, with the ratio of upregulated (red) to downregulated (blue) genes indicated for each term (Figure 3E-3F). Both GO and KEGG analyses revealed a striking convergence on lipid metabolism pathways. The most significantly enriched GO BP included "lipid biosynthetic process," "steroid biosynthetic process," "cholesterol biosynthetic process," and "fatty acid metabolic process" (Figure 3E). Similarly, KEGG analysis identified “Steroid biosynthesis,” “Terpenoid backbone biosynthesis,” “Biosynthesis of unsaturated fatty acids,” and “Fatty acid metabolism” among the toP enriched pathways (Figure 3F).

The consistency between GO and KEGG results strongly suggests that ES treatment induces substantial transcriptional reprogramming centered on lipid metabolism. Based on these findings, we hypothesize that disruption of lipid metabolic pathways may be a key mechanism underlying ES-induced cytotoxicity, and that IDH1-mutant cells may be particularly vulnerable to this metabolic stress.

### Defects in fatty acid metabolism exacerbate ES-induced cytotoxicity

**Figure 4:**
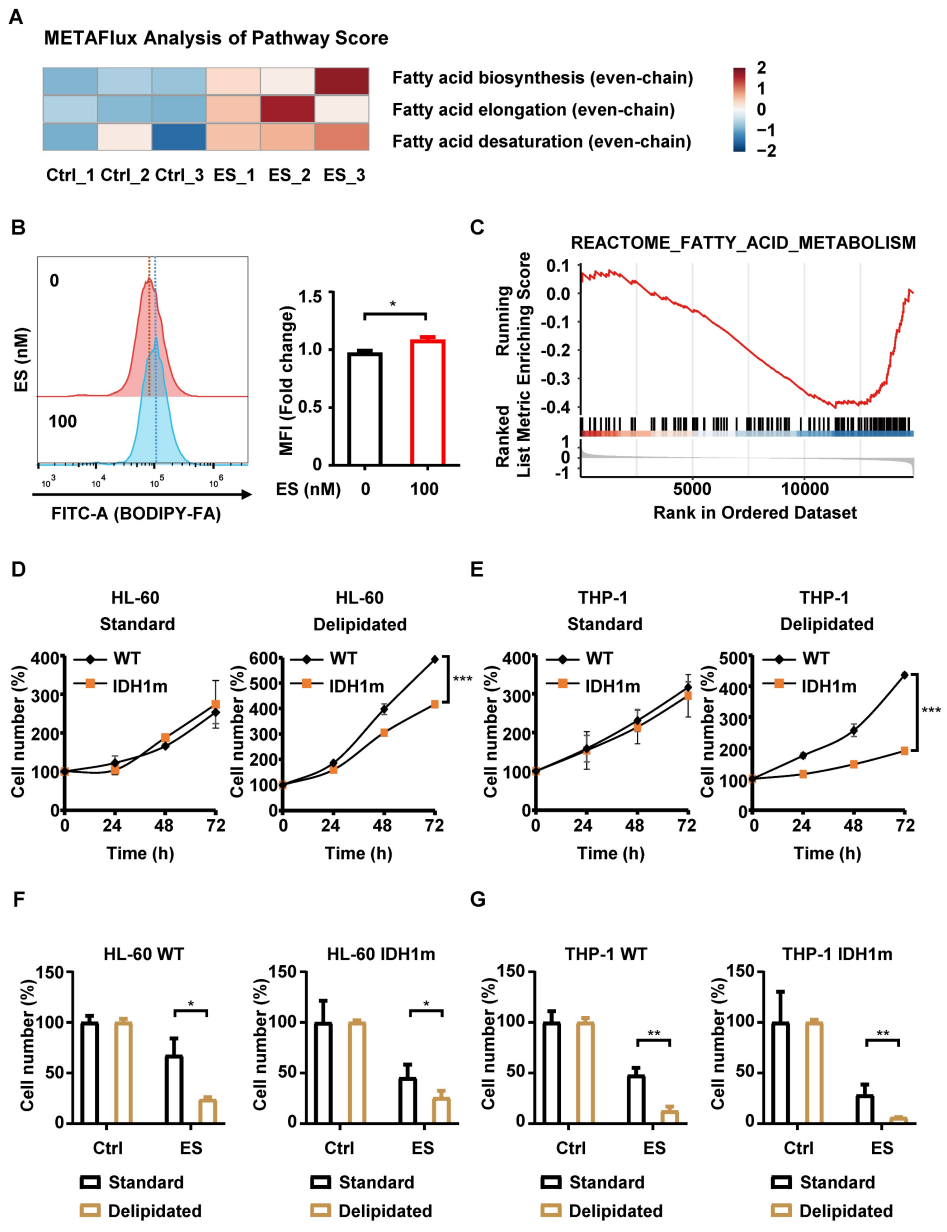
Defects in fatty acid metabolism exacerbate ES-induced cytotoxicity. (A) Metabolic flux analysis between the untreated grouP and the ES-treated grouP of the HL-60 cells, focusing on the pathways related to fatty acid (even-chain). (B) Measurement of fatty acid uptake in HL-60 cells with or without 100 nM ES treatment for 3 days by flow cytometry. (C) GSEA of DEGs comparing 19 AML samples with IDH1 mutations to 311 AML samples with wild-type IDH1 from the GSE24505 dataset, focusing on the enrichment of the “REACTOME_FATTY_ACID_METABOLISM” pathway. (D-E) HL-60/THP-1 WT or IDH1m cells were resuspended in either standard or delipidated medium and seeded. Time zero was defined at seeding; cck-8 reagent was added at 0, 24, 48 and 72 h, and absorbance was recorded. (F-G) HL-60/THP-1 WT or IDH1m cells were treated with or without 20 nM ES for 48 h in either standard or delipidated medium; cell number was assessed by cck-8 assay. (*, *P* < 0.05; **, *P* < 0.01; ***, *P* < 0.001.)

To quantify the activity of fatty acid-related pathways, we applied METAFlux, a transcriptome-based pathway scoring algorithm, to bulk RNA-seq data. We evaluated three sequential modules of even-chain fatty acid metabolism: *de novo* biosynthesis, elongation and desaturation. HeatmaP visualization revealed elevated flux scores for all three modules in ES-treated cells (Figure 4A), indicating increased dependency on fatty acid metabolic under ES exposure. This increased reliance likely extends to exogenous fatty acids. Supporting this, ES-treated HL-60 cells showed enhanced uptake of the fluorescent probe BODIPY-FA (Figure 4B), suggesting that ES promotes the assimilation of external lipids to meet metabolic demands.

Next, we investigated whether IDH1 mutation affects intrinsic fatty acid metabolism. Transcriptomic data from the GSE24505 dataset were analyzed to compare DEGs between 19 IDH1m and 311 WT AML patient samples. GSEA revealed negative enrichment of the “REACTOME_FATTY_ACID_METABOLISM” pathway in IDH1m samples (Figure 4C), suggesting a potential metabolic vulnerability.

To experimentally validate this, we cultured WT and IDH1m cells in RPMI-1640 medium supplemented with 10% delipidated fetal bovine serum (FBS), thereby restricting exogenous lipid availability. Under lipid-deprived conditions, IDH1m cell proliferation was significantly inhibited, whereas no significant differences were observed between WT and IDH1m cells in standard medium (Figure 4D-4E). These results indicate that IDH1m cells are more dependent on exogenous fatty acids for growth, implying an intrinsic defect in fatty-acid synthesis. Furthermore, both WT and IDH1m cells exhibited increased sensitivity to ES under lipid-deprived conditions (Figure 4F-4G), suggesting that an increased dependency on exogenous fatty acid uptake enhances cuproptosis.

Collectively, these findings demonstrate that ES increases cellular reliance on fatty acid metabolism, as evidenced by elevated BODIPY-FA uptake and synthetic lethality under lipid deprivation. Taken together, these results reveal a strong correlation between a dependency on exogenous fatty acids and heightened sensitivity to ES in IDH1-mutant cells. This suggests that the underlying defects in lipid metabolism may contribute to the selective vulnerability to cuproptosis, although the direct mechanistic link remains to be fully elucidated.

### ES more effectively inhibits HL-60 IDH1m tumor growth *in vivo*

**Figure 5:**
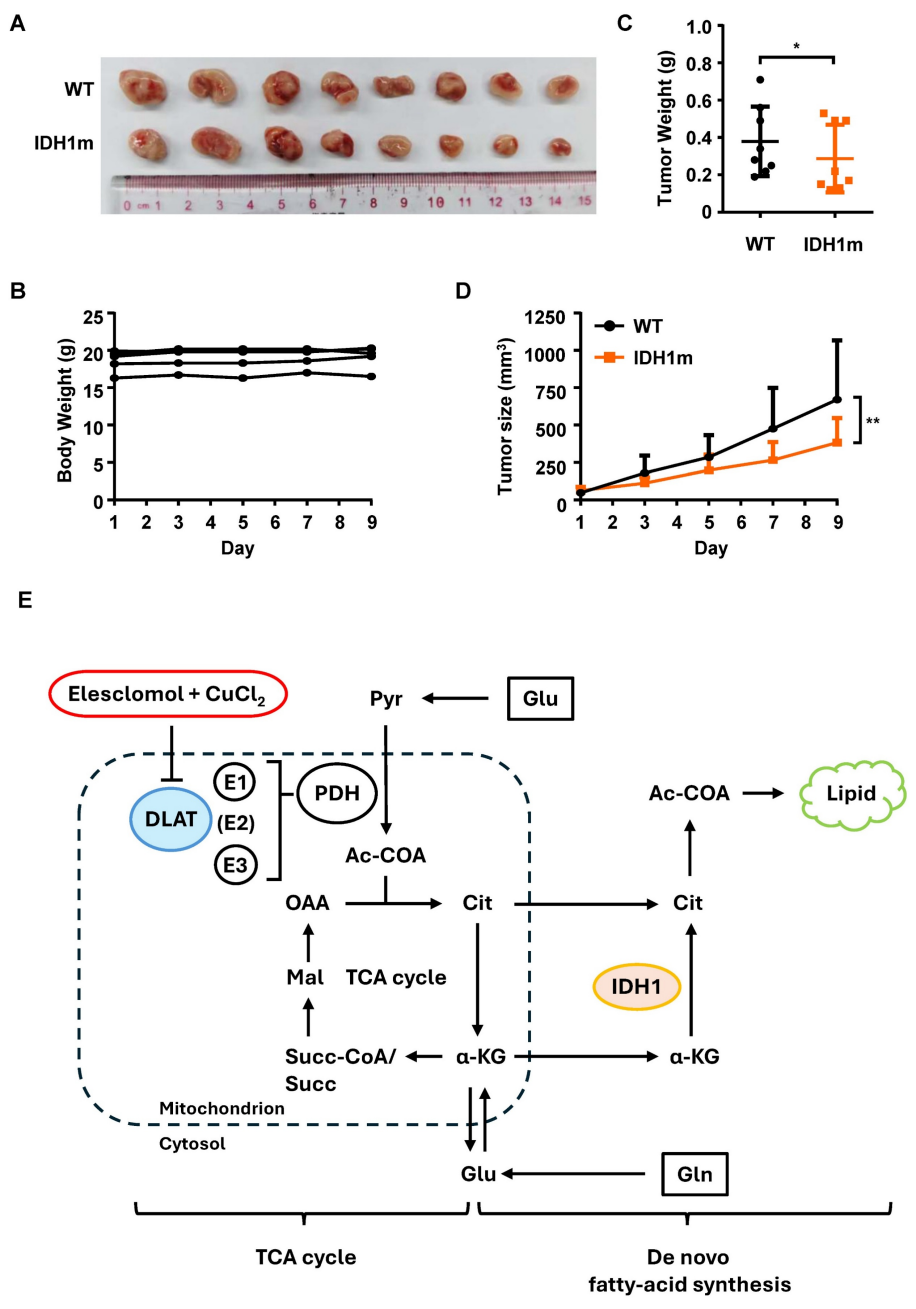
ES more effectively inhibits HL-60 IDH1m tumor growth than WT *in vivo*. (A) Images of HL-60 WT and HL-60 IDH1m tumors from ES-injected nude mice at the experimental endpoint (n= 8 independent animals). (B) The weight of tumor-bearing mice during ES treatment (20 mg/kg) via intraperitoneal (i.p.) injection every two days. (C) The weight of HL-60 WT and HL-60 IDH1m tumors from ES-injected nude mice at the experimental endpoint. (D) Tumor growth curve of HL-60 WT and HL-60 IDH1m tumors during ES treatment (20 mg/kg) via i.p. injection every two days. (E) Schematic illustration: ES suppresses DLAT activity, blocks the TCA cycle and activates glutamine-derived *de novo* fatty acid synthesis. (*, *P* < 0.05; **, *P* < 0.01.)

To minimize inter-animal variability, each nude mouse was implanted both HL-60 WT and HL-60 IDH1m cells in the left and right flanks, respectively. ES was administered intraperitoneally at a dose of 20 mg/kg every 2 days. At the experimental endpoint, tumors were harvested and arranged in paired comparisons (Figure 5A). Throughout the treatment period, mouse body weights remained stable, indicating good tolerability of ES (Figure 5B). Consistent with *in vitro* CCK-8 assay results, ES treatment significantly reduced tumor weights (Figure 5C) and volumes (Figure 5D) in HL-60 IDH1m xenografts compared to WT tumors. These *in vivo* findings corroborate the *in vitro* observations, confirming that IDH1m cells are more sensitive to ES-induced cytotoxicity.

## Discussion

We initially observed differences in cell viability between WT and IDH1m cells treated with ES under identical conditions. However, the oligomerization levels of DLAT were comparable at the protein level, suggesting that the sensitivity of IDH1m cells to ES could not be solely attributed to the mechanism of cuproptosis. Subsequently, we explored the mechanism underlying IDH1m cells' sensitivity to ES by considering both the functional alterations induced by ES treatment and the intrinsic functional differences between IDH1m and WT cells.

Published results indicate that cuproptosis relies on intact mitochondrial function, and mitochondrial dysfunction confers resistance to ES treatment 12. Given the functional inhibition of DLAT, we further explored the mechanism underlying IDH1m cells' sensitivity to ES from the perspective of PDC activity suppression. Theoretically, PDC inhibition would reduce TCA cycle flux and mitochondrial respiration. Experiments confirmed that ES treatment suppresses PDC activity and basal mitochondrial respiration rates. The suppression of basal respiration largely indicates the inhibition of oxidative phosphorylation, i.e., impaired mitochondrial energy metabolism, which is often compensated by the activation of glycolysis to meet cellular ATP demands. Experiments demonstrated that ES treatment increased lactate production and glucose consumption, hallmarks of glycolytic pathway activation, which further supports ES-induced mitochondrial energy metabolism impairment. Since ES-induced mitochondrial dysfunction is established, we questioned whether this effect would be more pronounced in IDH1m cells. Subsequently, MitoTracker and MitoSOX staining assays were designed in WT and IDH1m cells to preliminarily assess mitochondrial function. Results showed that ES effectively inhibited mitochondrial function in both cell types, yet no significant differences were observed between WT and IDH1m cells. Regarding the high-fluorescence peaks observed to the right of the main peak in the MitoSOX assay at 100 nM and 200 nM ES-treated grouP (Figure 2G-2H), a similar situation was described by Forkink et al. (2010). Due to the DNA-binding property of the MitoSOX dye, they observed that upon FCCP-induced dissipation of the mitochondrial membrane potential (Δψ), the MitoSOX dye translocates to the nucleus, producing a stronger nuclear fluorescence signal 26. Consistent with the increase in PI-positive cells with the treatment of 100 nM and 200 nM ES, we reasonably speculate that the collapse of Δψ associated with cell death at these conditions causes the MitoSOX probe to relocate to the nucleus, where DNA binding generates an intense, artifactual "false high-ROS peak." The results suggest that the mechanism of IDH1m sensitivity to ES cannot be fully explained by PDC activity suppression leading to reduced TCA cycle flux and mitochondrial dysfunction.

To explore mechanisms beyond the known suppression of mitochondrial function that might underlie the heightened sensitivity of IDH1m cells to ES, we performed RNA-sequence on ES-treated HL-60 cells. Integrating the toP 20 terms from both GO and KEGG pathway enrichment analysis, we observed a dominant and consistent enrichment of lipid-related pathways among the most significantly perturbed terms. This convergence prompted us to formulate a new hypothesis: ES broadly impairs cellular lipid-metabolism networks, thereby imposing a state of lipid-metabolic deficiency, and IDH1m cells are particularly vulnerable to this deficiency, a vulnerability that may explain their exaggerated sensitivity to ES. We also acknowledge that our transcriptomic analysis was performed exclusively in wild-type cells. While this allowed us to define the general pathways affected by ES, a future direct transcriptomic comparison between wild-type and IDH1-mutant cells following treatment would undoubtedly provide a more comprehensive understanding of the differential response and is an important direction for future investigation. Experimentally confirmed, IDH1m cells were more reliant on exogenous fatty acids for growth compared to WT cells, and a lipid-depleted environment further sensitized cells to ES. Thus, it is reasonable to infer that the heightened sensitivity of IDH1m cells to ES is due to their intrinsic dependence on exogenous fatty acids. We acknowledge that the fatty acid uptake assay used in this study primarily reflects the cells' reliance on external lipid sources and may not be a direct proxy for global metabolic flux. Future studies, such as isotopic tracing and metabolic flux analysis, would be required to more precisely delineate these global changes.

Regarding the mechanism by which ES induces a global lipid-metabolic defect, we noted that it has been reported that in PDHA1-silenced cells, the primary pathway for de novo fatty acid synthesis shifts from the oxidative TCA cycle fueled by glucose to the reductive TCA cycle initiated by glutamine (Figure S1H- S1I), with enhanced uptake of exogenous fatty acids. Moreover, PDHA1-silenced cells exhibit intrinsic growth defects compared to WT, which can be partially rescued by fatty-acid supplementation 27.

Since the direct mechanism of ES is to inhibit PDC activity, the findings from this study are consistent with our observation of synergistic lethality between ES treatment and deprivation of exogenous fatty acids. Additionally, the mentioned study shows that when the PDHA1 gene is silenced, cells are forced to use the reductive TCA cycle (which starts with glutamine) to make new fatty acids. A key enzyme in this cycle is IDH1. Our study began with the observation that cells with an IDH1 mutation are more easily killed by ES. This study highlights the need to consider the intrinsic impairment of the reductive TCA cycle by IDH1 mutation when explaining the sensitivity to ES. So, for IDH1m cells, fatty acid metabolism, and ES toxicity, we now have a possible explanation: IDH1m cells have a broken reductive TCA cycle compared to normal cells, so they can't make fatty acids well through this path. When treated with ES, which forces the cell to depend mainly on the reductive TCA cycle for making fatty acids, the IDH1 mutation means the cell can't meet this new demand. This makes the IDH1m cells much more sensitive to ES.

Collectively, the mechanisms underlying the heightened sensitivity of IDH1m cells to cuproptosis are illustrated (Figure 5E). DLAT, as the E2 subunit of PDC, becomes inactivated upon ES induction, directly leading to the suppression of overall PDC activity. This prevents the conversion of pyruvate to acetyl-CoA, thereby depriving the substrates of TCA cycle and ultimately resulting in mitochondrial dysfunction. The second part shows that silencing of PDC activates the reductive TCA cycle initiated by glutamine, which becomes the primary pathway for de novo fatty acid synthesis. Faced with the shift toward the reductive TCA cycle caused by impaired PDC activity, IDH1m cells may be unable to meet the resulting metabolic demand due to their inherently lower reductive TCA flux compared to WT cells, a consequence of their defective IDH1 enzyme which is normally crucial for sustaining this pathway.

In summary, the heightened vulnerability of IDH1-mutant cells to ES is, at least in part, attributable to an inherent defect in lipogenesis. The concomitant triggering of cuproptosis and the crippling of fatty acid anabolism may therefore constitute a synthetic-lethal partnership. Beyond IDH1-mutant malignancies, this copper-dependent metabolic collateral vulnerability offers a conceptual framework for therapeutic exploitation across broader pathological contexts.

## Materials and Methods

### Reagents

The following reagents were used in this study: Elesclomol (HY-12040) was obtained from MCE (NJ, USA). Copper (II) chloride (CuCl2; 751944) and Ammonium tetrathiomolybdate (TTM; 323446) were obtained from Sigma Aldrich (MO, USA). Delipidated fetal bovine serum (MP20222) was obtained from Shanghai Yuanye Bio-Technology (Shanghai, China). Propidium Iodide (PI) was obtained from the Annexin V-FITC Apoptosis Detection Kit I (556547, BD Bioscience, CA, USA). Mito-Tracker DeeP Red FM (C1032) and BODIPY 500/510 C1, C12 (Fatty Acid Green Fluorescence Probe; C2055) were obtained from Beyotime (Shanghai, China). MitoSOX Red Mitochondrial Superoxide Indicator (M36008) was obtained from Invitrogen (MA, USA). For all experiments described in this study, both in vitro and in vivo, ES was used in combination with CuCl₂ at a 1:1 molar ratio to ensure effective copper delivery and induction of cuproptosis.

### Cell culture

Human AML cell line HL-60 was obtained from the US Model Culture Collection (ATCC, Rockville, USA). Human AML cell line THP-1 was obtained from Chinese Academy of Sciences Cell Bank (Shanghai, China). These cell lines were cultured in RPMI-1640 medium (Gibco, New York, USA), which was supplemented with 10% fetal bovine serum (FBS; Gibco, New York, USA) and 1% Pen StreP Glutamine (100×, 10000 Units/ml penicillin, 10 mg/ml streptomycin). The cells were cultured at 37 °C in a humidified incubator with 5% CO_2_.

### Construction of stable cell lines

THP-1 cell lines over-expressing wild-type IDH1 (WT) or the R132H mutant (IDH1m) were previously established in our laboratory. The IDH1-WT construct was derived from the pLenti-CMV-IDH1-GFP-Puro plasmid (Public Protein/Plasmid Library, PPL01050-4a); the IDH1R132H mutant plasmid was generated by site-directed mutagenesis as pLenti-CMV-IDH1R132H-GFP-Puro. HL-60 WT and HL-60 IDH1m lines were produced by identical lentiviral protocol. Briefly, HEK293T cells were co-transfected with the IDH1 or IDH1R132H expression plasmid together with psPAX2 and pMD2.G in OPTI-MEM medium (Gibco, New York, USA) using FuGENE HD (Promega, WI, USA) according to the manufacturer's instructions. After 16 h, the medium was replaced with fresh full medium. Viral supernatants were harvested at 24 h post-transfection. For lentiviral infection by spinoculation, HL-60 cells were resuspended in viral supernatant with 8 µg/ml polybrene (Sigma, MO, USA), centrifuged at 800× g for 1 h at 37 °C, incubated overnight, then expanded in complete RPMI-1640 medium. After 48 h, transduced HL-60 cells were selected with puromycin (400 µg/ml) for generation HL-60 WT and HL-60 IDH1m.

### Cell counting kit-8 assay

The Cell Counting Kit-8 (cck-8; Meilunbio, Dalian, China) was used to measure cell proliferation. Cells were maintained at 1 × 10⁴ cells in 96-well plates with a total volume of 100 µL per well. At the endpoint of experiments, 5 µL of cck-8 solution was added to each well, followed by incubation at 37 °C for 2 h. Absorbance was then measured at 450 nm using the SpectraMax iD5 Multi-Mode Microplate Reader (Molecular Devices, CA, USA). For drug-sensitivity assay of ES, ES was added at the time of seeding cells at concentrations of 0, 5, 10, 20, 40, and 80 nM. After 48 h of culture, cell number was assessed by cck-8 assay. For the time-course cell proliferation assay, RPMI-1640 supplemented with 10 % FBS—used for routine culture—was designated “standard 1640 medium”, whereas RPMI-1640 containing 10 % delipidated FBS (MP20222, Shanghai Yuanye Bio-Technology, Shanghai, China) was termed “delipidated 1640 medium”. The time point at which cells were seeded was designated as 0 h; cck-8 reagent was added at 0, 24, 48 and 72 h to access cell proliferation.

### Flow cytometric analysis of dead-cell quantification by Propidium Iodide (PI)

HL-60 WT, HL-60 IDH1m, THP-1 WT and THP-1 IDH1m cells were treated with ES (0, 100, 200 nM) for 48 h, then the cells were harvested. PI, obtained from the Annexin V-FITC Apoptosis Detection Kit I (556547, BD Bioscience, CA, USA), was used to stain cells in accordance with the manufacturer's recommendation. The fluorescence of PI within the cells was then measured using the PE-A channel by flow-cytometry (Beckman Coulter, CA, USA).

### Western blotting

For ES dose-response, HL-60/THP-1 WT cells were treated with ES (0, 50, 100, 200 nM) for 48 h. For single-dose assay of ES, HL-60/THP-1 WT or IDH1m cells were treated ± 200 nM ES for 48 h. Cells were rinsed with ice-cold PBS, lysed in 1% SDS radioimmunoprecipitation assay (RIPA) buffer containing protease and phosphatase inhibitors (Roche, Basel, Switzerland) on ice for 0.5 h. Lysates were clarified (12000× g, 15 min, 4 °C), and protein was quantified with the BCA Protein Assay Kit (P0012, Beyotime, Shanghai, China). Equal amounts (20 µg) were mixed with 5× loading buffer (10% β-mercaptoethanol), heated (95 °C, 5 min) and resolved on 10 % SDS-PAGE gels. Proteins were transferred onto polyvinylidene difluoride (PVDF) membranes (Millipore, MA, USA) pre-wetted with methanol. Membranes were blocked in 5 % Bovine Serum Albumin (BSA) prepared from Tris-buffered saline with Tween 20 (TBST) for 1 h, incubated overnight at 4°C with primary antibodies (dilutions per manufacturer) in 5 % BSA prepared from TBST, washed (3× 5 min, TBST) and probed with horseradish peroxidase (HRP)-conjugated secondary antibodies (1:10000, 1 h, RT). Bands were revealed using SuperSignal West Pico Chemiluminescent Substrate (Thermo Scientific, MA, USA) and imaged on the Amersham ImageQuant 800 system (Cytiva, MA, USA). β-actin was used as the loading control. Primary antibodies for immunoblotting were purchased from the following sources: DLAT (4A4-B6-C10) Mouse mAb (#12362), Pyruvate Dehydrogenase (C54G1) Rabbit mAb (PDHA1; #3205) were from Cell signaling technology (MA, USA). Anti-Lipoic Acid antibody (ab58724), Anti-PDHA1 (phospho S293) antibody (ab177461) were from Abcam (MA, USA). HSP70 (3A3) (sc-32239) was from Santa Cruz (CA, USA). Mouse monoclonal anti-β-actin (#A5441) anti-body was from Sigma Alarich (MO, USA).

### PDC activity measurement

HL-60/THP-1 WT or IDH1m cells were treated with ± 200 nM ES for 48 h, then the cells were harvested. The PDC activity assay mixture for cellular samples was prepared strictly according to the manufacturer's instructions provided with Pyruvate Dehydrogenase Kit (ab287837, Abcam, MA, USA). Using the SpectraMax iD5 Multi-Mode Microplate Reader (Molecular Devices, CA, USA) in 120-min kinetic mode at 37 °C, the enzymatic conversion of substrate to product by PDC in cell lysates was monitored; increased PDC activity was reflected by higher product accumulation at 450 nm.

### Oxygen consumption rate (OCR) measurement

HL-60/THP-1 WT or IDH1m cells were treated with ± 200 nM ES for 24 h, then the cells were harvested. Cells were resuspended as 1× 10^7^ cells/ml in the 37 °C pre-warmed medium. As mentioned previously, OCR was quantified with a Fluorescence Lifetime Micro Oxygen Monitoring System from Instech Instrument (Plymouth, PA, USA) at a controlled constant temperature of 37 °C. Cell suspensions (330 µL) were transferred to the chamber containing the oxygen-sensitive probe, and the oxygen was recorded every 10 s since the chamber was sealed. OCR (% O_2_ min⁻¹) was derived from the linear slope of the oxygen decay curve and normalized to the protein content of the cells.

### Glucose uptake and lactate secretion assays

HL-60/THP-1 WT cells were treated with or without 100 nM ES for 3 days, then the cells and the cell supernatant were collected. Using Glucose Assay Kit with O-toluidine (S0201, Beyotime, Shanghai, China) and L-Lactic Acid (LA) Colorimetric Assay Kit (E-BC-K044-S, Elabscience Biotechnology, Wuhan, China) to detect the concentration of glucose and lactate in the culture medium in line with the manufacturer's guidelines, respectively. The standard culture medium was used as the base value. Protein content of the cells was used for normalization.

### Flow cytometric analysis of Mitochondrial ROS

HL-60/THP-1 WT or IDH1m cells were treated with ES (0, 100, 200 nM) for 48 h, then the cells were collected. For the measurement of reactive oxygen species (ROS) in mitochondria, MitoSOX Red Mitochondrial Superoxide Indicator (M36008, Invitrogen, MA, USA) was used to stain cells in accordance with the manufacturer's recommendation. The fluorescence of MitoSOX Red Mitochondrial Superoxide Indicator (MitoSox) within the cells was then measured using the PC7-A channel by flow-cytometry (Beckman Coulter, CA, USA).

### Flow cytometric analysis of Mito-Tracker DeeP Red FM

The processing method of the samples was as described above. Mito-Tracker DeeP Red FM (C1032, Beyotime, Shanghai, China) selectively labels metabolically active mitochondria and accumulates in these organelles in a membrane-potential-dependent manner; thus, it also serves as an indicator of mitochondrial membrane potential. For the measurement of mitochondrial membrane potential, Mito-Tracker DeeP Red FM (MitoT) was used to stain cells in accordance with the manufacturer's recommendation. The fluorescence of MitoT within the cells was then measured using the APC-A channel by flow-cytometry (Beckman Coulter, CA, USA).

### Transcriptomic analysis

HL-60 cells were divided into two groups: untreated control and 200 nM ES-treated for 48 h (n = 3 each). Cell samples were sent to Shanghai Bioprofile Biotechnology Co., Ltd. for transcriptomic analysis. Total RNA of cells was extracted with TRIzol, quantified by NanoDrop. mRNA was poly-A-selected, Illumina-fragmented, reverse-transcribed (SuperScript II), second-strand synthesized (DNA Pol I/RNase H), end-repaired, 3′-adenylated, PE-adaptor-ligated, AMPure-purified (400-500 bp), 15-cycle PCR-enriched, QC-analysed (Bioanalyzer) and sequenced on NovaSeq Xplus. The RNA-seq data were analyzed using R software (version 4.1.0). Differential gene expression analysis was performed using the “limma” package. In the differential expression analysis, genes were considered significantly regulated if they met the criteria of an absolute log2(Fold Change) ≥ 1 and a P-value < 0.05. Gene set enrichment analysis (GSEA), Gene Ontology (GO) and Kyoto Encyclopedia of Genes and Genomes (KEGG) enrichment analysis were conducted using the “clusterProfiler” package 28. The estimation of metabolic flux from bulk RNA-seq data was performed using the “METAFlux” package (available at https://github.com/KChen-lab/METAFlux), which was employed to calculate activity for pathways related to fatty acid metabolism 29.

### Flow cytometric analysis of fluorescently labeled fatty acid uptake

BODIPY 500/510 C1, C12 (Fatty Acid Green Fluorescence Probe; Biyotime, C2055, Shanghai, China) was used to quantify fatty-acid uptake according to the manufacturer's protocol. HL-60 cells were treated with or without 100 nM ES for 3 days, then the medium was replaced with the delipidated 1640 medium. After 24 h, BODIPY 500/510 C1, C12 (Fatty Acid Green Fluorescence Probe; BODIPY-FA) was added to a final concentration of 1 µM and the cells were incubated at 37 °C for 3 h before harvesting. The fluorescence of BODIPY-FA within the cells was then measured using the FITC-A channel by flow-cytometry (Beckman Coulter, CA, USA).

### Bioinformatic analysis of GSE24505 dataset

The RNA-seq data from GSE24505 dataset were obtained from the Gene Expression Omnibus (GEO) database (available at https://www.ncbi.nlm.nih.gov/geo/). For dataset GSE24505, RNA-seq data from AML patient samples with wild-type IDH1 (311 cases) and with IDH1 mutations (19 cases) were analyzed.^5^ The RNA-seq data were analyzed using R software (version 4.1.0). Differential gene expression analysis was performed using the “limma” package, and gene set enrichment analysis (GSEA) was conducted using the “clusterProfiler” package.^28^

### Xenograft assay

All animal experiments were conducted in line with the guidelines by the Division of Animal Control and Inspection of the Department of Food and Animal Inspection and Control of Macau and were approved by the Animal Care and Use Committee of the Macau University of Science and Technology. We made every effort to minimize the number of animals used in each experimental grouP and to reduce animal suffering.

Eight female BALB/c nude mice (6 weeks) were anesthetized with isoflurane. A suspension of 1 × 10^7^ HL-60 WT cells in 100 µL PBS was injected subcutaneously into the left flank; the corresponding HL-60 IDH1m cells were implanted at the same density into the right flank. When tumors reached ~50 mm^3^, ES (20 mg/kg) was administered intraperitoneally every 2 days for the entire study. Body weight and tumor dimensions (length and width) were measured every 2 days. Tumor volume was calculated as (width^2^ × length)/2. Animals were euthanized when any tumor exceeded 1000 mm^3^.

### Statistical analysis

Statistical analysis was conducted in GraphPad Prism 9. All values are expressed as mean ± SD. Differences between the groups were tested using two-tailed unpaired Student's t-tests (*, *P* < 0.05; **, *P* < 0.01; ***, *P* < 0.001; ns, *P* > 0.05.).

## Supplementary Material

Supplementary figure.

## Data Availability

All data generated or analyzed during this study are included in this published article. Data related to this article can be obtained from the corresponding author on request.

## Abbreviations

2-HG: 2-Hydroxyglutarate
α-KG: α-Ketoglutarate
AML: Acute Myeloid Leukemia
ATP: Adenosine Triphosphate
BP: Biological Process
BSA: Bovine Serum Albumin
CCK-8: Cell Counting Kit-8
CSCs: Cancer Stem Cells
DEGs: Differentially Expressed Genes
DLAT: Dihydrolipoamide S-Acetyltransferase
ES: Elesclomol
Fe-S: Iron-Sulfur
FBS: Fetal Bovine Serum
GO: Gene Ontology
GSEA: Gene Set Enrichment Analysis
HSP70: Heat Shock Protein 70
IDH1: Isocitrate Dehydrogenase 1
IDH1m: R132H-Mutant IDH1
KEGG: Kyoto Encyclopedia of Genes and Genomes
lipo-DLAT: Lipoylated DLAT
i.p.: Intraperitoneal
MitoSOX: MitoSOX Red Mitochondrial Superoxide Indicator
MitoT: Mito-Tracker DeeP Red FM
OAA: Oxaloacetate
PCA: Principal-component analysis
PDC: Pyruvate Dehydrogenase Complex
PVDF: Polyvinylidene Difluoride
RIPA: Radioimmunoprecipitation Assay
ROS: Reactive Oxygen Species
TBST: Tris-Buffered Saline with Tween 20
TCA: Tricarboxylic Acid
TTM: Ammonium Tetrathiomolybdate
WT: Wild-Type
ΔΨ: Membrane Potential
