# Supplementary figures and images for: IDH1 mutation creates a dependency on fatty acid metabolism that underlies sensitivity to cuproptosis in acute myeloid leukemia cells
Source: Int J Med Sci. 2026 Feb 26;23(4):1243–56. doi: 10.7150/ijms.127886 (PMC13048861; doi:10.7150/ijms.127886)

Supplementary Material

A

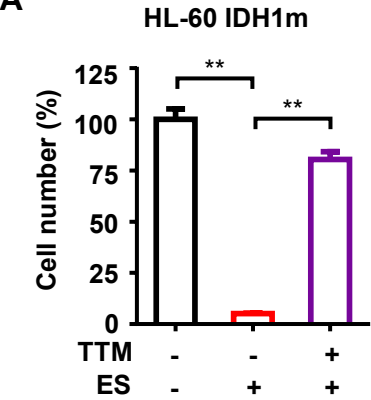

B

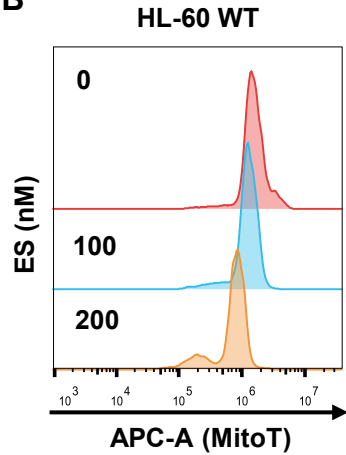

C

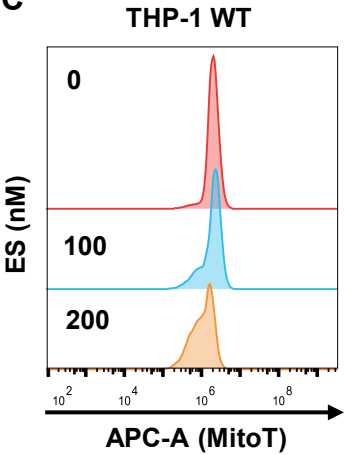

D

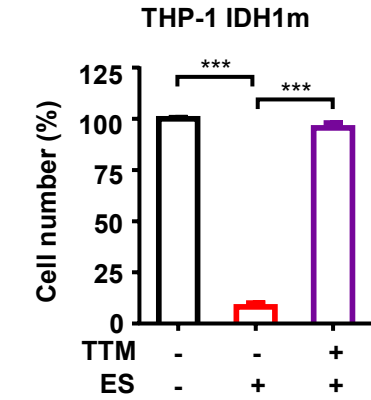

E

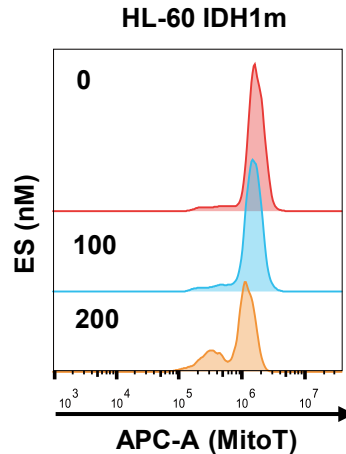

F

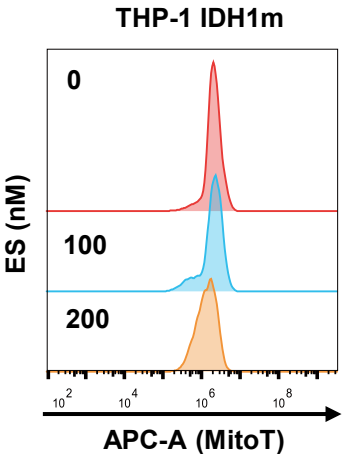

G

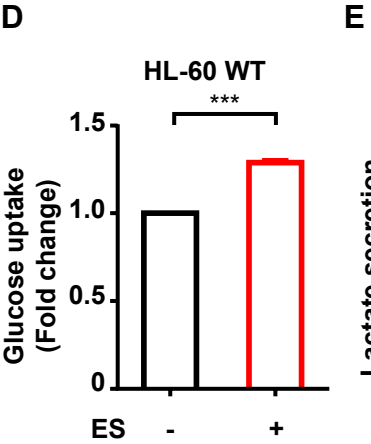

H

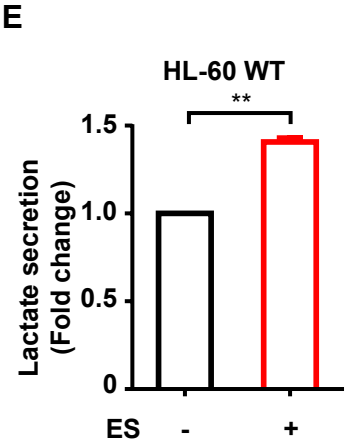

I

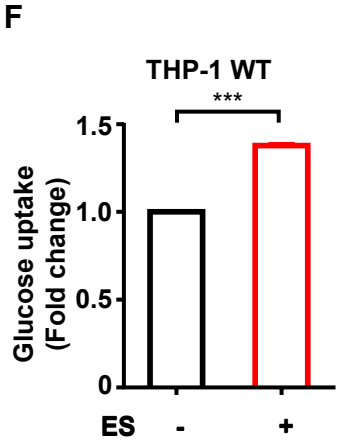

J

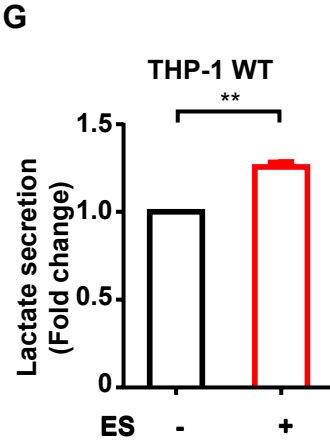

K

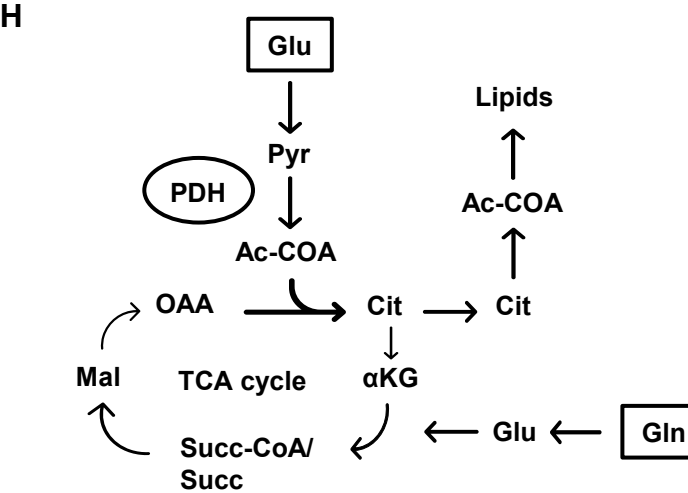

L

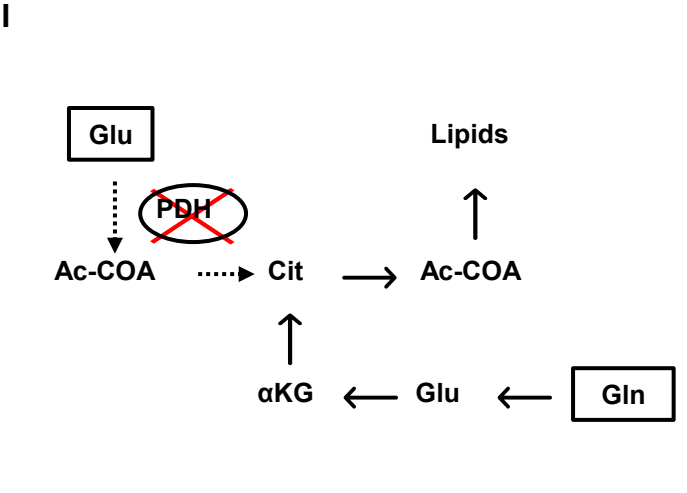

Supplement: Supplementary file 1 — Supplementary figure. [file ijmsv23p1243s1.pdf]
